# FIAN: A frequency information-adaptive network for spatial-frequency domain pansharpening

**DOI:** 10.1371/journal.pone.0324236

**Published:** 2025-06-03

**Authors:** Yang Liu, Wei Wang, Weihe Li

**Affiliations:** 1 School of Computer Science and Technology, Wuhan University of Science and Technology, Wuhan, China; 2 Hubei Province Key Laboratory of Intelligent Information Processing and Real-Time Industrial System, Wuhan University of Science and Technology, Wuhan, China; 3 Wuhan Huamao Automation Co., LTD., Wuhan, China; Prince Mohammad Bin Fahd University, SAUDI ARABIA

## Abstract

Pansharpening aims to combine the spatial information from high-resolution panchromatic (PAN) images with the spectral information from low-resolution multispectral (LRMS) images generating high-resolution multispectral (HRMS) images. While Convolutional Neural Networks (CNNs) have shown impressive performance in pansharpening tasks, their tendency to focus more on low-frequency information will lead to suboptimal preservation of high-frequency details, which are crucial for producing HRMS images. Recent studies have highlighted the significance of frequency domain information in pansharpening, but existing methods often consider the network as a whole, overlooking the unique abilities of different layers in capturing high-frequency components. This oversight can result in the loss of fine details and limit the overall performance of pansharpening. To overcome these limitations, we propose FIAN, a novel frequency information-adaptive network designed specifically for spatial-frequency domain pansharpening. FIAN introduces an innovative frequency information-adaptive filter module that can dynamically extract frequency-domain information at various frequencies, enabling the network to better capture and preserve high-frequency details during the pansharpening process. Furthermore, we have developed a frequency feature selection strategy to accurately extract the most relevant frequency-domain information, enhancing the network’s representational power. Lastly, we present a multi-frequency information fusion module that effectively combines the frequency-domain information extracted by the filter at different frequencies with the spatial-domain information. We conducted extensive experiments on multiple benchmark datasets to evaluate the effectiveness of the proposed method. The experimental results demonstrate that our approach achieves competitive performance compared to state-of-the-art pansharpening methods.

## Introduction

High-resolution multispectral (HRMS) images are vital across a range of sectors, including agriculture, industry, and civil infrastructure. Due to the limitations of hardware equipment, HRMS cannot be directly acquired. Pansharpening overcomes this obstacle by effectively fusing high-resolution panchromatic (PAN) images with low-resolution multispectral(LRMS) images, thus synthesizing HRMS images [8, 76], providing better data support for the subsequent analysis and application of remote sensing images [2, 7, 12, 14, 20, 63].

Masi *et al*. [26] developed and trained PNN, the pioneering pansharpening network based on convolutional neural networks (CNNs), which employs a three-layer convolution structure. The success of PNN has spurred extensive research into CNN-based pansharpening methods, leading to substantial advancements in the field [3, 9, 29, 31, 33, 34, 37, 40–42, 45, 46].

**Fig 1:**
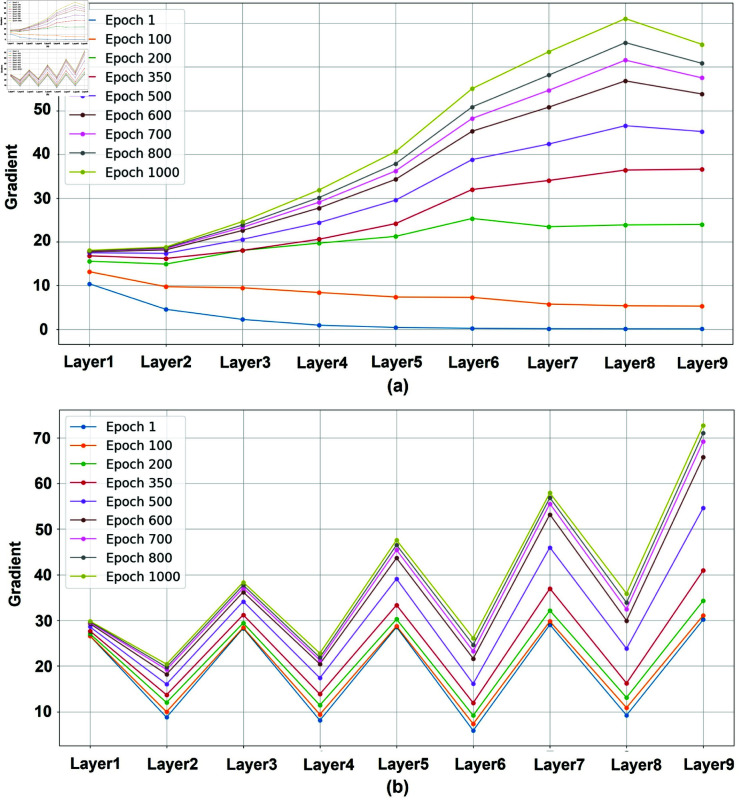
Gradients of network features. Distribution of discrete gradients of network features at different training stages. (a): The evolution of discrete gradient values for each layer in the network as training iterations (epochs). (b): The changes in discrete gradient values for network layers with skip connections throughout the training iterations (epochs).

Further investigation into the characteristics of CNN layers reveals notable frequency-related properties. As shown in Fig 1(a), we perform a frequency analysis on the features extracted by each layer of a representative nine-layer CNN at various stages of training. This study employs the discrete gradient as a measure to assess the frequency composition of layer features; larger gradient values suggest an increased proportion of high-frequency components.

Fig 1(a) shows that during the initial phase of training (e.g., epoch=1), the gradients of shallow layer features substantially exceed those of deeper layers, indicating that the CNN primarily captures low-frequency image content. As training progresses (for example, epoch = 350), the gradients of the features of the deep layer gradually increase, eventually exceeding those of the shallower layers. This suggests that CNN begins to capture high-frequency details. By Epoch 1000, the gradients of deep layer features significantly outweigh those of shallow layers, implying that deeper layers predominantly focus on high-frequency image information. This observation highlights the frequency-selective nature of CNNs, exhibiting a transition from low-frequency to high-frequency feature extraction. Consequently, this frequency bias impacts the performance of pansharpening in two ways: firstly, it leads to a slower convergence rate of CNNs in the pansharpening task; secondly, the emphasis on low-frequency information in shallow layers may result in the loss of crucial high-frequency details during feature extraction.

To mitigate these challenges, researchers have explored the incorporation of skip connections, such as in DiCNN [50]. Fig 1(b) depicts the distribution characteristics of the finite difference gradients across CNN layers after introducing skip connections. It is evident that odd and even layers exhibit distinct gradient distribution patterns, collectively forming a “W" shape. This suggests that skip connections modulate the frequency extraction behavior of CNNs to a certain degree. By propagating the output features of the current layer along with those of the previous layer to the subsequent layer, skip connections help alleviate the loss of high-frequency information that arises from the limited high-frequency extraction capability of the current layer. However, closer examination reveals that within odd and even layer groups, the frequency extraction characteristics still align with those observed in Fig 1(a). This implies that while skip connections provide some relief for high-frequency loss, they do not fundamentally resolve the frequency extraction bias inherent to CNNs [22].

In an effort to further enhance pansharpening performance, researchers have begun to investigate methods that synergize frequency and spatial domain information. However, existing approaches often treat CNNs as monolithic entities, disregarding the varying capacities of different network layers in extracting high-frequency information. Naively fusing frequency domain information with spatial domain features may fail to provide the most appropriate frequency components for the current CNN layer, exacerbating the loss of high-frequency details and compromising the effectiveness of dual-domain information fusion.

To tackle these challenges, we introduce FIAN, an innovative frequency information-adaptive network designed specifically for spatial-frequency domain pansharpening. At the heart of FIAN lies the frequency information-adaptive filter module, which intelligently extracts vital frequency-domain information for features at various network layers by leveraging their gradient information. Additionally, we have developed a frequency feature selection strategy that judiciously extracts particular frequency information based on the gradient distribution of features. Lastly, to seamlessly merge the frequency-domain information at different frequencies with the spatial-domain information, we present a multi-frequency information fusion module, which significantly enhances the performance of pansharpening. Comprehensive evaluations performed on remote sensing image datasets highlight the exceptional performance of our proposed approach when contrasted with the most advanced pansharpening methods currently available.

The primary contributions of this paper are as follows:

- We introduce a novel frequency information-adaptive filter module that intelligently extracts essential frequency-domain information for features at different network layers by leveraging their gradient information.
- We develop a judicious frequency feature selection strategy that selectively extracts specific frequency information based on the gradient distribution of features.
- We present a multi-frequency information fusion module to seamlessly integrate the frequency-domain information at different frequencies with the spatial-domain information.

## Related work

In recent decades, researchers have extensively explored pansharpening, leading to the development of four primary methods [23]: Component Substitution (CS) [30], Multiresolution Analysis (MRA) [35], Variational Optimization (VO), and Deep Learning (DL). CS methods [15, 16, 19, 67, 68] focus on substituting the spatial component of LRMS images with the detailed spatial information from PAN images, aiming to preserve the original spectral content to the greatest extent within the transform domain. MRA techniques [8, 10, 11, 35] involve decomposing LRMS images into multi-resolution components and enhancing them with the high-frequency, detail-rich aspects of PAN images. VO approaches [53, 55–58] leverage prior knowledge to formulate constraints that guide the model, achieving pansharpening through an optimized algorithmic solution.

Pansharpening methodologies grounded in deep learning have captured widespread attention and marked significant advancements. Convolutional neural networks (CNNs), renowned for their exceptional proficiency in extracting both spectral and spatial information, have ascended to a pivotal role in pansharpening research [5, 6, 29, 31, 33, 34, 37, 39–42, 44–46]. Consequently, scholars have been delving into diverse strategies to augment the efficacy of pansharpening techniques within the spatial domain. However, despite the commendable performance of CNNs in this realm, they still grapple with challenges in capturing high-frequency details [22], often culminating in high-resolution multispectral images (HRMS) with inadequate texture details [22]. To mitigate this limitation, some researchers have begun to pivot towards frequency domain methods [13, 24, 38, 43, 49]. These approaches can more adeptly capture and preserve high-frequency information, thereby complementing the limitations inherent in spatial domain techniques. By extracting and amalgamating information within the frequency domain, researchers aspire to elevate the overall effectiveness of pansharpening, thus achieving superior image detail restoration throughout the pansharpening process.

### CNN-based pansharpening methods

Masi *et al* [26]. pioneered this domain with the CNN-based PNN method, which employs a convolutional network to extract spatial features from PAN and LRMS images, subsequently integrating them into the upsampled LRMS image. However, the relatively simple architecture of PNN, consisting of merely three convolutional layers, results in slower convergence speeds. To address this, the DiCNN [50] method introduces skip connections between various layer feature maps, thereby mitigating gradient explosion and accelerating network convergence. Nevertheless, its simplistic network structure and limited number of parameters constrain its feature extraction capabilities. Zhang *et al* [44]. proposed the BDPN method, which enhances the accuracy of multi-scale detail information extraction in multispectral images. BDPN, inspired by traditional MRA methods, leverages a pyramid-structured bidirectional network to process both LRMS and PAN images. However, the extensive parameter size of BDPN complicates network training and limits the model’s generalization ability. MSDCNN [39] adopts a different strategy, utilizing two branches to extract information from both deep and shallow image layers. Each branch employs three convolution kernels of varying sizes to capture multi-scale features. By integrating features from different receptive fields, MSDCNN improves feature extraction accuracy. However, the versatility of its convolution kernels introduces increased uncertainty in learning deep and shallow features. FusionNet [51], another notable advancement, estimates a nonlinear injection model for detailed information using a deep network structure. It directly processes the original upsampled multispectral and pan images for differential detail extraction. The extracted details are then refined through multiple residual network blocks for further feature extraction and learning. The final output, when added to the upsampled multispectral image, yields the fusion image. This approach, while innovative, contends with the challenge of managing the complex interactions between different network layers.

### Dual-domain pansharpening methods based on spatial and frequency domains

In light of advancements in CNN-based spatial domain pansharpening methods, researchers have explored incorporating frequency domain information to further enhance the quality of fused HRMS images. Man Zhou *et al*. [24] proposed a pansharpening method that fuses both domains, designing the Spatial-Frequency Information Integration Network (SFIIN), which extracts local spatial features from PAN and LRMS images while integrating frequency domain information to enhance their global context. This dual-domain fusion significantly boosts pansharpening performance. Subsequently, Yuan *et al*. [13] constructed the Pyramid-based Dual-Domain Network (PYDDN), injecting multi-scale frequency domain spatial details from the PAN image into multi-scale spatial information through a frequency feature pyramid. Hou *et al*. [38] proposed the BIM method, comprising two branches: band-aware local specificity modeling and Fourier global detail reconstruction. The local specificity modeling uses adaptive convolution kernels to process local discrepancies across spectral bands, while the global detail reconstruction branch leverages Fourier domain global modeling capabilities to recover lost global details. BIM exhibits exceptional local-to-global representation learning ability through the combination of spatial and frequency domains. Furthermore, Zhou *et al*. [43] introduced the Spatial-Frequency Information Integration Network (SFINet) and its improved version, SFINet++. The core component of SFINet, SFIB, fuses a spatial domain branch for processing local information, a frequency domain branch capturing global information using discrete Fourier transform, and a learning mechanism facilitating cross-domain information interaction. SFINet++ achieves significant performance improvements by incorporating lossless information fusion via reversible neural operators. Zhang *et al*. [49] proposed the Wavelet Domain Network (WINet), leveraging wavelet domain processing for efficient pansharpening. The Wavelet-Inspired Fusion Block (WFB) in WINet aims to achieve lossless information fusion through inter-subband interaction, while the High-frequency Enhancement Block (HEB) integrates subband information to enhance high-frequency features. By combining wavelet and CNN techniques, WINet effectively synthesizes high-quality HRMS images from spatial and frequency domain information. Lastly, Wang *et al*. [54] introduced an innovative pansharpening architecture where the Feature Extraction and Enhancement Module (FEM) utilizes fast Fourier convolution and attention mechanisms to form a hybrid of global and local receptive fields in the high-frequency domain. The Implicit Neural Alignment (INA) aligns multi-scale high-frequency features through accurate implicit neural representations, while the Pre-align Module develops an efficient trainable upsampling operator to address the inherent alignment challenge between PAN and MS images.

The significance of integrating frequency domain information with spatial domain features in dual-domain pansharpening methods is increasingly evident. This integration enables the production of high-quality HRMS images. By leveraging the complementary strengths of both spatial and frequency domains, these approaches demonstrate superior performance compared to traditional CNN-based spatial domain methods. However, existing methodologies often consider the CNN network in its entirety when integrating dual-domain information, overlooking the fact that different layers within a CNN network process frequency domain information of varying frequencies. This oversight potentially hampers the quality of dual-domain information fusion. Treating the CNN network as a monolith for frequency domain information fusion may not fully harness the capabilities of each layer, resulting in suboptimal dual-domain information fusion. To improve the effectiveness of dual-domain information fusion, we introduce FIAN, an innovative frequency information-adaptive network designed specifically for spatial-frequency domain pansharpening.

## Materials and methods

**Fig 2:**
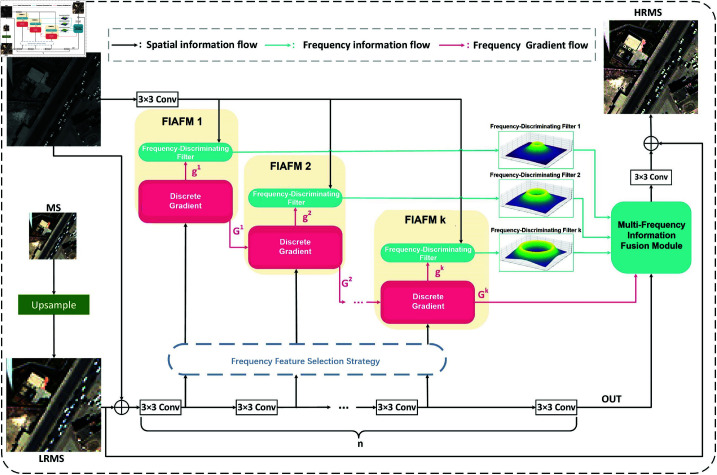
The overall framework of FIAN. The overall framework of the suggested frequency information-adaptive network for spatial-frequency domain pansharpening(FIAN). FIAFM stands for frequency information-adaptive filter module. Reprinted from https://github.com/liangjiandeng/PanCollection under a CC BY license, with permission from Liangjian Deng, original copyright 2025.

Fig 2 presents the overall framework of FIAN. The process begins by upsampling a multispectral (MS) image to create a low-resolution multispectral (LRMS) image. This LRMS image, combined with a panchromatic (PAN) image, is fed into a convolutional neural network (CNN). In this framework, we propose a frequency information-adaptive filter module to extract frequency information from different features.

The frequency information-adaptive filter module computes the discrete gradient of the CNN features, which, together with the PAN features, directs the filter extraction. These filters exhibit varying frequencies due to differences in the layer outputs. Subsequently, the frequency feature selection strategy identifies suitable features based on the gradient range. The frequency information-adaptive filter module extracts the frequency information from the selected features. The frequency information at various frequencies and their corresponding gradient calculations are then processed in the multi-frequency information fusion module. To mitigate the gradient vanishing problem commonly observed in convolutional neural networks, we have incorporated the Rectified Linear Unit (ReLU) activation function after every CNN layer in our network architecture.

### Frequency information-adaptive filter module

The filter generation process in the frequency information-adaptive filter module comprises two crucial stages: the computation of discrete gradients from the features and the subsequent creation of frequency-discriminating filters corresponding to these gradients.

#### Discrete gradient.

To calculate the discrete gradients of CNN features, we apply a sequence of mathematical computations. Let $F^{i}\in {\mathbb{R}}^{B\times C\times H\times W}$ be the feature tensor of the *i*-th selected layer of the CNN, where *B*, *C*, *H*, and *W* denote the batch size, number of channels, height, and width of the feature maps, respectively. The gradient computation process for $F^{i}$ is as follows:

First, we calculate the absolute differences between adjacent pixels in both horizontal and vertical directions to obtain the gradient tensors ${g^{i}}_{x}$ and ${g^{i}}_{y}$:

$${𝐠}_{x}^{i}=\left|{𝐅}^{i}[:,:,:,:-1]-{𝐅}^{i}[:,:,:,1:]\right|\;{𝐠}_{y}^{i}=\left|{𝐅}^{i}[:,:,:-1,:]-{𝐅}^{i}[:,:,1:,:]\right|$$ (1)

Here, ${𝐠}_{x}^{i}$ has dimensions [*B*, *C*, *H*, (*W*–1)] and ${𝐠}_{y}^{i}$ has dimensions [*B*, *C*, (*H*–1), *W*].

To ensure the gradient tensors have the same dimensions as the original feature tensor, we apply zero-padding to ${𝐠}_{x}^{i}$ and ${𝐠}_{y}^{i}$:

$${𝐠}_{x}^{i}=\text{pad}({𝐠}_{x}^{i},(0,1,0,0))\;{𝐠}_{y}^{i}=\text{pad}({𝐠}_{y}^{i},(0,0,0,1))$$ (2)

After padding, both ${𝐠}_{x}^{i}$ and ${𝐠}_{y}^{i}$ have dimensions [*B*, *C*, *H*, *W*]. Next, we compute the gradient magnitude using the Euclidean norm:

$${𝐌}^{i}=\sqrt{{𝐠}_{x}^{i2}+{𝐠}_{y}^{i2}}$$ (3)

where ${𝐌}^{i}$ has dimensions [*B*, *C*, *H*, *W*]. Finally, we perform global average pooling on the gradient magnitude tensor ${𝐌}^{i}$ to obtain the mean of the scalar gradient ${𝐠}^{i}$:

$${𝐠}^{i}=\frac{1}{BCHW}\sum_{b=1}^{B}\sum_{c=1}^{C}\sum_{h=1}^{H}\sum_{w=1}^{W}{𝐌}^{i}[b,c,h,w]$$ (4)

The gradient computation process for the features of the *i*-th selected layer is summarized as:

$${𝐠}^{i}({𝐅}_{BCHW}^{i})=\frac{1}{BCHW}\sum_{b,c,h,w}\sqrt{{\left(P_{x}(\left|\Delta_{x}{𝐅}^{i}\right|)\right)}^{2}+{\left(P_{y}\left(\left|\Delta_{y}{𝐅}^{i}\right|\right)\right)}^{2}}$$ (5)

where $\Delta_{x}{𝐅}^{i}$ and $\Delta_{y}{𝐅}^{i}$ represent the computation process in Eq 1, while *P*_*x*_ and *P*_*y*_ represent the padding process in Eq 2.

The computed value ${𝐠}^{i}$ represents the gradient magnitude of the current CNN feature. A higher value of ${𝐠}^{i}$ indicates a higher frequency of information present in the network feature. By examining the variations in ${𝐠}^{i}$ values across different network layers, this approach quantitatively directs the extraction process of the frequency-discriminating filter.

Fig 2 depicts the discrete gradient calculation procedure utilized in the frequency information-adaptive filter components. The gradient computation result for the *i*-th module is denoted by ${𝐠}^{i}$. The aggregated gradient result from the previous (*i* – 1)-th module is represented by ${𝐆}^{i-1}$, while the aggregated result passed to the next (i  +  1)-th module is represented by ${𝐆}^{i}$. The mathematical formulation of ${𝐆}^{i}$ is provided in Eq 6:

$${𝐆}^{i-1}=[{𝐠}^{1},{𝐠}^{2},\cdots ,{𝐠}^{i-1}]\;{𝐆}^{i}=[{𝐠}^{1},{𝐠}^{2},\cdots ,{𝐠}^{i-1},{𝐠}^{i}]$$ (6)

This expression represents the process of gathering discrete gradient information layer by layer during the network’s forward pass. ${𝐆}^{i-1}$ is a vector that aggregates the gradient computation results from the first frequency information-adaptive filter module up to the (*i* – 1)-th module. ${𝐆}^{i}$ is obtained by appending the gradient computation result ${𝐠}^{i}$ of the current layer to the end of ${𝐆}^{i-1}$.

#### Frequency-discriminating filter.

This study presents an innovative frequency-discriminating filter that adaptively captures frequency information from the current feature based on its gradient ${𝐠}^{i}$.

In particular, the cutoff frequency ${𝐃}_{i}\in {\mathbb{R}}^{B\times C}$ of the frequency-discriminating filter is defined as *k* times the gradient ${𝐠}^{i}$, expressed as ${𝐃}_{i}=k$ × ${𝐠}^{i}$. Furthermore, we introduce an enhancement factor α and a decay factor β. These factors are employed to modify the upper and lower cutoff frequencies of the frequency-discriminating filter, respectively, as elaborated in Eq 7:

$${𝐃}_{i}^{enhance}=\alpha \times {𝐃}_{i}\;{𝐃}_{i}^{decay}=\beta \times {𝐃}_{i}$$ (7)

Here, ${𝐃}_{i}^{enhance}\in {\mathbb{R}}^{B\times C}$ and ${𝐃}_{i}^{decay}\in {\mathbb{R}}^{B\times C}$ represent the enhanced upper cutoff frequency and the decayed lower cutoff frequency, respectively.

We develop a frequency-discriminating filter ${FD}^{i}\in {\mathbb{R}}^{B\times C\times H\times W}$. Eq 8 presents the mathematical expression of the filter ${FD}_{b,c}^{i}$ for each batch and channel:

$${FD}_{b,c}^{low,i}(u,v)=\sqrt{1+{\left(\frac{D(u,v)}{{𝐃}_{i}^{decay}(b,c)}\right)}^{2}}-1\;{FD}_{b,c}^{high,i}(u,v)=2-\sqrt{1+{\left(\frac{D(u,v)}{{𝐃}_{i}^{enhance}(b,c)}\right)}^{2}}\;{FD}_{b,c}^{i}(u,v)={FD}_{b,c}^{low,i}(u,v)\times {FD}_{b,c}^{high,i}(u,v)$$ (8)

Where *D*(*u*, *v*) represents the Euclidean distance from the point (*u*, *v*) to the center of the spectrum, defined by the formula $D(u,v)=\sqrt{(u-\frac{H}{2}{)}^{2}+(v-\frac{W}{2}{)}^{2}}$. Aggregating ${FD}_{b,c}^{i}$ across all batches and channels results in ${FD}^{i}\in {\mathbb{R}}^{B\times C}$.

The two-dimensional Fast Fourier Transform (FFT) is first performed on the PAN image, as shown in Eq 9. Each PAN image $PAN\in {\mathbb{R}}^{B\times C\times H\times W}$ can be viewed as a collection of *B* three-dimensional tensors with dimensions (*C*, *H*, *W*). For the two-dimensional image ${PAN}_{b,c}\in {\mathbb{R}}^{H\times W}$ in the *c*-th channel of the *b*-th batch, a two-dimensional FFT is applied to obtain its spectrum ${𝐅}_{PAN}^{b,c}$.

$${𝐅}_{PAN}^{b,c}=FFT({PAN}_{b,c})$$ (9)

Next, the amplitude spectrum ${𝐀}_{PAN}^{b,c}$ and phase spectrum ${𝐏}_{PAN}^{b,c}$ are extracted separately, as shown in Eq 10:

$${𝐀}_{PAN}^{b,c}=|{𝐅}_{PAN}^{b,c}|\;{𝐏}_{PAN}^{b,c}=∠{𝐅}_{PAN}^{b,c}$$ (10)

where | · | and ∠· represent the modulus and argument of a complex number, respectively. The amplitude spectrum ${𝐀}_{PAN}^{b,c}\in {\mathbb{R}}^{H\times W}$ represents the intensity of each frequency component, while the phase spectrum ${𝐏}_{PAN}^{b,c}\in {\mathbb{R}}^{H\times W}$ contains the phase information of each frequency component.

After performing the Fourier transform on all batches and channels of the images, we obtain the representation of the PAN image in the frequency domain. The amplitude spectrum tensor ${𝐀}_{PAN}\in {\mathbb{R}}^{B\times C\times H\times W}$ and the phase spectrum tensor ${𝐏}_{PAN}\in {\mathbb{R}}^{B\times C\times H\times W}$ are composed of all ${𝐀}_{PAN}^{b,c}$ and ${𝐏}_{PAN}^{b,c}$, respectively.

Finally, the ${FD}^{i}$ is multiplied element-wise with the amplitude spectrum ${𝐀}_{PAN}$ to obtain the filtered amplitude spectrum ${𝐀}_{filtered}^{i}\in {\mathbb{R}}^{B\times C\times H\times W}$ and the phase spectrum tensor ${𝐏}_{filtered}^{i}$, as shown in Eq 11:

$${𝐀}_{filtered}^{i}={𝐀}_{PAN}⊙{FD}^{i}\;{𝐏}_{filtered}^{i}={𝐏}_{PAN}⊙{FD}^{i}$$ (11)

The following is a concise summary of the frequency-discriminating filter process.

$$\left({𝐀}_{filtered}^{i},{𝐏}_{filtered}^{i})=FDF(FFT(PAN),{FD}^{i}({𝐠}^{i},k,\alpha ,\beta )\right)$$ (12)

The coefficients *k*, α, and β are empirically determined to be 5.0, 1.5, and 0.8, respectively.

The frequency-discriminating filter introduced in this work adaptively modifies the cutoff frequencies according to the discrete gradients derived from the network layer features. This adaptive approach fully leverages the characteristics of the current network layer features, enabling the effective extraction of frequency domain information from PAN images within the appropriate range. Consequently, this enhances the accuracy and adaptability of high-frequency detail extraction.

### Frequency feature selection strategy

As shown in Fig 1, the gradient information contained in the features of each CNN layer varies, reflecting information from the frequency domain at different frequencies. Existing pansharpening methods that combine spatial and frequency domains have not fully considered this characteristic. Instead, they treat the CNN network as a whole for frequency domain information fusion, which makes it difficult to fully exploit the unique advantages of each network layer, thereby affecting the performance of dual-domain information fusion.

**Fig 3:**
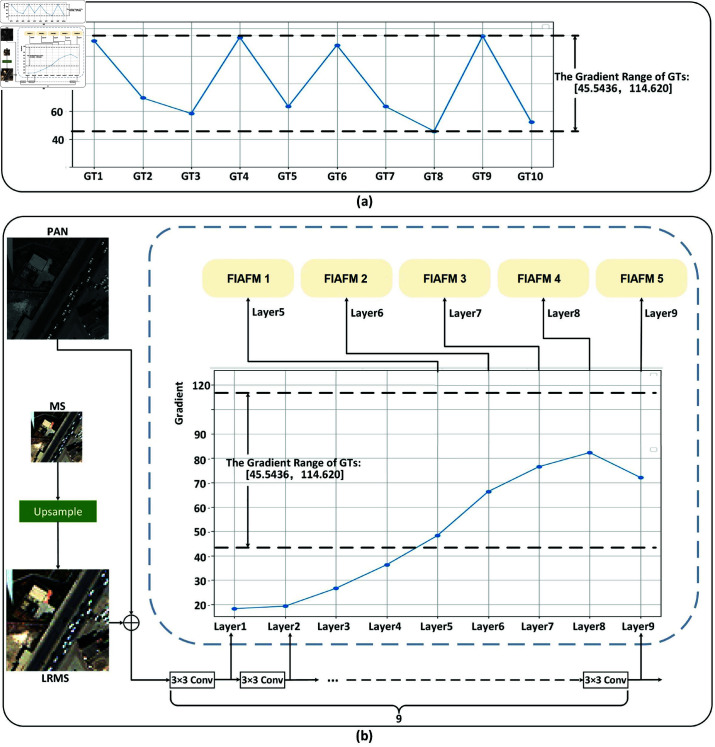
Schematic diagram of the frequency feature selection strategy. Schematic illustration of the frequency feature selection approach. (a) The spectrum of discrete gradients for ten randomly chosen GT data. (b) The procedure of identifying features that correspond to the numerical range of the GT discrete gradients and subsequently inputting these selected features into the frequency information-adaptive filter module for additional processing. FIAFM stands for frequency information-adaptive filter module. Reprinted from https://github.com/liangjiandeng/PanCollection under a CC BY license, with permission from Liangjian Deng, original copyright 2025.

To thoroughly analyze this problem, we randomly selected 10 samples from the training data and calculated the gradients of their ground truth (GT) images. The results indicate that the gradient range of the GT images is 45.5436-114.6202, as shown in Fig 3(a). Correspondingly, we performed the same gradient calculation on the features of a nine-layer CNN network. The gradient range of the nine-layer network features is 18.1816-82.3504, and as the network layers deepen, the feature gradients exhibit an upward trend. This suggests that when extracting image features, the CNN network prioritizes low-frequency information, which may lead to the loss of high-frequency details during training.

To mitigate the issue of high-frequency information loss, we earlier proposed a frequency information-adaptive filter module. This filter extracts information from different frequency ranges for different network layers and fuses it with spatial domain features. However, for certain CNN network layers (such as the first layer), the feature gradients are relatively low (only 18.1816), limiting the extracted frequency information. Moreover, this low-frequency information can already be well-extracted by the CNN network. Accordingly, this paper puts forward a frequency feature selection strategy founded on feature gradient intervals to enhance the network’s capacity for representation.

As depicted in Fig 3(b), the gradient values of the network layer features reveal that the frequency information contained in layers five through nine displays a higher degree of correspondence to the frequency information present in the GT. This indicates that the frequency information contained within the network layer features from the fifth to the ninth layers substantially affects the high-frequency details of the generated HRMS images. Consequently, we utilize this frequency information to refine and enhance the intricate high-frequency components of the HRMS images. This targeted feature selection strategy is designed to better supplement and enhance high-frequency details while preserving low-frequency information, ultimately improving the overall performance of dual-domain information fusion. We refer to this process as the range matching of network feature gradients, which involves aligning the numerical span of gradients derived from the CNN features with those obtained from the ground truth data.

### Multi-frequency information fusion module

**Fig 4:**
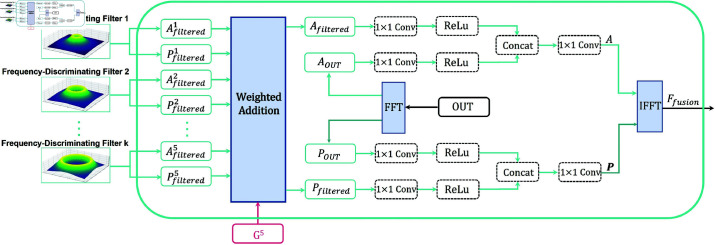
Multi-frequency information fusion module. The multi-frequency information fusion module amalgamates the results from a quintet of distinct frequency-discriminating filters with the feature output derived by the CNN network. In the schematic diagram, “OUT" represents the output features of the CNN network. FFT stands for Fast Fourier Transform, while IFFT represents the Inverse Fourier Transform.

The multi-frequency information fusion module, depicted in Fig 4, integrates the amplitude spectra ${𝐀}_{filtered}^{i}$ and phase spectra ${𝐏}_{filtered}^{i}$ obtained from five distinct frequency-discriminating filters using a weighted combination, as formulated in Eq 13.

$${𝐀}_{filtered}=\sum_{i=1}^{5}w_{i}\cdot {𝐀}_{filtered}^{i}\;{𝐏}_{filtered}=\sum_{i=1}^{5}w_{i}\cdot {𝐏}_{filtered}^{i}$$ (13)

The normalization of the discrete gradient ${𝐆}^{5}=[g^{1},g^{2},g^{3},g^{4},g^{5}]$ yields the weight vector $𝐖=[w_{1},w_{2},w_{3},w_{4},w_{5}]$, as detailed in Eq 14:

$$w_{i}=\frac{g^{i}}{\sum_{j=1}^{n}g^{j}}$$ (14)

The weighted fusion yields the amplitude spectrum ${𝐀}_{filtered}$ and phase spectrum ${𝐏}_{filtered}$. Simultaneously, we perform a fast Fourier transform (FFT) on output features of the CNN network name OUT to obtain its amplitude spectrum ${𝐀}_{OUT}$ and phase spectrum ${𝐏}_{OUT}$, a process previously described in Eq 9 and Eq 10.

Next, the fused amplitude spectrum ${𝐀}_{filtered}$, phase spectrum ${𝐏}_{filtered}$, and the OUT’s amplitude spectrum ${𝐀}_{OUT}$ and phase spectrum ${𝐏}_{OUT}$ are respectively fed into two 1×1 convolution, and the ReLU activation function is applied to the convolved features. Then, the two sets of activated amplitude spectrum features and phase spectrum features are concatenated separately, and finally, a 1×1 convolution is used to obtain the fused amplitude spectrum **A** and phase spectrum **P**.

Lastly, the fused amplitude spectrum **A** and phase spectrum **P** are input into the inverse Fourier transform (IFFT) to obtain the spatial-domain multi-frequency band fusion feature ${𝐅}_{fusion}$.

The multi-frequency information fusion module integrates data from various frequency ranges by performing a weighted combination of the amplitude and phase spectra, along with the spectral information of the LRMS image.

## Result analysis and discussion

In this section, we shall evaluate the proposed method and juxtapose its results with those of recent, competitive approaches. The data employed in these evaluations originate from datasets procured from the WorldView-3 (WV3), WorldView-2 (WV2), and QuickBird (QB) satellites. Initially, we will delineate the specifics of the datasets utilized for this analysis in greater detail. Following this, we will outline the evaluation metrics and the particulars of the training process. Subsequently, we will exhibit the test results of the proposed method alongside those of the comparative methods across the three datasets, incorporating both quantitative metrics and visual illustrations under low-resolution and full-resolution conditions, to affirm the effectiveness of the proposed FIAN. Ultimately, the efficacy of the suggested FIAN and its component modules will be meticulously examined via an extensive series of ablation studies.

### Dataset

To evaluate our pansharpening method, we used an extensive dataset, including 4-band datasets from QB and 8-band datasets from WV3 and WV2. The 4-band sets include standard colors: red, green, blue, and near-infrared; the 8-band sets add coastal, yellow, red-edge, and an additional near-infrared band. The spatial resolution ratio between PAN (panchromatic) and MS (multispectral) images is 4:1. Owing to the absence of ground truth images, we spatially degraded the datasets using the Wald’s protocol [47], which involves downsampling the original images by a factor of 4 through filters aligned with each satellite’s modulation transfer function (MTF). In our network, the simulated datasets’ panchromatic and multispectral images serve as inputs, while the original multispectral images act as reference data for training. The datasets used in this study are from the PanCollection dataset [17], which contains detailed data descriptions. For example, in the WV3 dataset, we used 9,714 pairs of PAN-LRMS-GT data for network training, where the PAN images have a size of 64×64×1, the LRMS images have a size of 16×16×8, and the GT images have a size of 64×64×8. For low-resolution evaluation, we selected 20 pairs of PAN-LRMS-GT data for testing, where the PAN images have a size of 256×256×1, the LRMS images have a size of 64×64×8, and the GT images have a size of 256×256×8. For full-resolution evaluation, we selected 20 pairs of PAN-LRMS images for testing, where the PAN images have a size of 512×512×1 and the LRMS images have a size of 128×128×8.

### Training details and parameters

In this study, we developed our network using the PyTorch 2.0 framework on a Linux system, with a single NVIDIA GeForce GTX 3090 GPU supporting our hardware needs. Our software setup also included Python 3.10. Throughout the training phase, we conducted 1000 epochs. The learning rate started at 3 × 10^−4^ for the initial 500 epochs, then reduced to one-tenth of that rate for the latter half. We utilized the ADAM optimizer [52] to manage parameter optimization, maintaining a batch size of 32 and setting $\beta_{1}$ at 0.9 and $\beta_{2}$ at 0.999.

### Comparison methods and quantitative metrics

To assess the performance of our proposed method, we performed both qualitative and quantitative comparisons against current state-of-the-art pansharpening techniques. We compared our method with two main types of approaches: CNN-based spatial domain methods (including PNN [26], MSDCNN [39], DRPNN [31], DiCNN [50], BDPN [44], FusionNet [51] and dual-domain methods that integrate spatial and frequency domains (including WINet [49], PYDDN [13], SFINet [43], BIM [38]). All competing methods were trained on identical training sets, using parameters specified in their respective original publications. To ensure fairness and consistency in our evaluations, all methods were tested under the same hardware and software conditions.

This paper provides a comprehensive assessment of the performance of the methods mentioned earlier, using both reduced-resolution and full-resolution datasets. For the reduced-resolution tests, we employ several metrics: ERGAS [21], SAM [4], SCC [32], and Q2n [36]. These metrics help quantify the quality of the results. For the full-resolution tests, we use the $D_{\lambda }^{\left(K\right)}$, *D*_*S*_, and QNR [1] to evaluate the performance of all methods.

### Performance comparison with WV3 data

In this section, we conduct a comprehensive evaluation and comparison of the proposed method and benchmark methods on the WV3 dataset, considering both reduced-resolution and full-resolution aspects.

First, we assess the differences between the predicted pansharpening images and the ground truth (GT) images through reduced-resolution evaluation. Table 1 presents the objective assessment metric results for all compared methods and our proposed model across 20 WV3 test samples. The table demonstrates that our model achieves the best performance across all metrics, showcasing the effectiveness and superiority of our approach. Compared to benchmark pansharpening methods, our model better preserves the spectral information of multispectral images while effectively enhancing spatial resolution, resulting in high-quality image fusion.

**Table 1:** Quantitative metrics of reduced-resolution WV3 data.

Method	SAM	ERGAS	SCC	Q2n
PNN [26]	4.5100±1.2039	3.6262±1.2759	0.9388±0.0303	0.8154±0.1259
MSDCNN [39]	4.5443±1.1549	3.7156±1.2977	0.9356±0.0317	0.7962±0.1023
BDPN [44]	4.6275±1.7874	4.0892±1.3620	0.9310±0.0316	0.7965±0.1126
DRPNN [31]	4.4196±1.2029	3.6377±1.2685	0.9348±0.0310	0.8119±0.0944
DiCNN [50]	4.7126±1.4394	3.7208±1.4191	0.9348±0.0339	0.8192±0.0827
FusionNet [51]	4.2269±1.0480	3.1124±1.0296	0.9247±0.0413	0.8105±0.0893
WINet [49]	3.2948±0.4460	2.3978±0.2703	0.9777±0.0002	0.8540±0.0095
SFINet [43]	3.3012±0.3879	2.4264±0.2050	0.9771±0.0103	0.8502±0.0093
PYDDN [13]	3.3004±0.3579	2.3540±0.2368	0.9763±0.0124	0.8554±0.0119
BIM [38]	3.1921±0.3691	2.2632±0.2145	0.9795±0.0094	0.8564±0.0099
FIAN(ours)	3.1281±0.3099	2.2179±0.1884	0.9798±0.0082	0.8572±0.0097
Ideal value	0	0	1	1

Average quantitative with the related standard deviations (std) comparisons on 20 reduced-resolution WorldView-3 examples, with the best performance highlighted in red.

**Fig 5:**
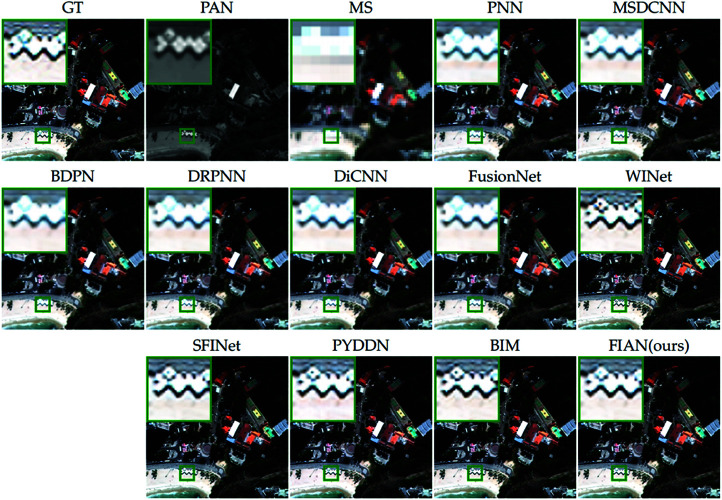
Visual comparisons of reduced-resolution WV3 data. The visual comparisons of fusion results from various methods applied to reduced-resolution data from the WorldView-3 satellite. Reprinted from https://github.com/liangjiandeng/PanCollection under a CC BY license, with permission from Liangjian Deng, original copyright 2025.

Fig 5 provides a visual comparison of the pansharpening results from all compared methods to further illustrate the performance differences. We selected a representative area of the image (marked with a green box), enlarged it, and positioned it in the upper left corner of the original image for detailed observation. Close examination of the enlarged area reveals that the fusion results produced by our model closely resemble the ground truth image, exhibiting excellent performance in texture, edges, and color reproduction.

Secondly, we assess the pansharpening performance of various methods on actual data through full-resolution evaluation. Table 2 displays the objective assessment metric results for all compared methods and our proposed model across 20 full-resolution WV3 test samples. The results in the table demonstrate that our model achieves the best performance across all metrics, highlighting the effectiveness and superiority of our approach.

**Table 2:** Quantitative metrics of full-resolution WV3 data.

Method	QNR	$D_{\lambda }^{\left(K\right)}$	$D_{S}$
PNN [26]	0.8921±0.0552	0.0629±0.0336	0.0489±0.0248
MSDCNN [39]	0.7847±0.1328	0.1407±0.0885	0.0867±0.0677
BDPN [44]	0.7905±0.1035	0.1368±0.0687	0.0774±0.0579
DRPNN [31]	0.8192±0.1164	0.1166±0.0730	0.0741±0.0587
DiCNN [50]	0.8446±0.0714	0.1127±0.0586	0.0433±0.0237
FusionNet [51]	0.8570±0.0913	0.0863±0.0495	0.0622±0.0519
WINet [49]	0.9366±0.0351	0.0359±0.0289	0.0263±0.0173
SFINet [43]	0.9212±0.0456	0.0314±0.0250	0.0572±0.0393
PYDDN [13]	0.9262±0.0469	0.0285±0.0185	0.0551±0.0377
BIM [38]	0.9354±0.0385	0.0201±0.0110	0.0457±0.0286
LABPFFN(ours)	0.9426±0.0272	0.0185±0.0101	0.0413±0.0244
Ideal value	1	0	0

Average quantitative metrics across 20 full-resolution samples from the WorldView-3 dataset are presented, with the best performance highlighted in red.

**Fig 6:**
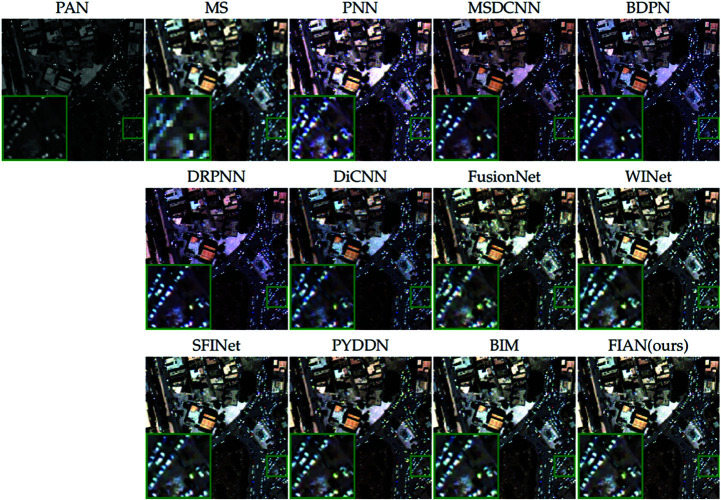
Visual comparisons of full-resolution WV3 data. The visual comparisons of fusion results from various methods applied to full-resolution data from the WorldView-3 satellite. Reprinted from https://github.com/liangjiandeng/PanCollection under a CC BY license, with permission from Liangjian Deng, original copyright 2025.

Fig 6 provides a visual comparison of the pansharpening results from all methods evaluated, further illustrating performance differences. We selected a representative area of the image (marked with a green box), enlarged it, and placed it in the lower left corner of the original image for detailed observation. Upon close examination of the enlarged area, it is evident that the fusion results produced by our model excel in texture, edge definition, and color reproduction.

### Performance comparison with WV2 data

In this section, we conduct a comprehensive evaluation and comparison of the proposed method and benchmark methods on the WV2 dataset, considering both reduced-resolution and full-resolution aspects.

First, we assess the differences between the predicted pansharpening images and the ground truth (GT) images through reduced-resolution evaluation. Table 3 presents the objective assessment metric results for all compared methods and our proposed model across 20 WV2 reduce-resolution test samples. The table demonstrates that our model achieves the best performance across all metrics, showcasing the effectiveness and superiority of our approach. Compared to benchmark pansharpening methods, our model better preserves the spectral information of multispectral images while effectively enhancing spatial resolution, resulting in high-quality image fusion.

**Table 3:** Quantitative metrics of reduced-resolution WV2 data.

Method	SAM	ERGAS	SCC	Q2n
PNN [26]	9.5862±1.4418	7.5088±0.1820	0.9112±0.0410	0.7506±0.1007
MSDCNN [39]	8.9993±0.5488	7.6409±0.1227	0.9127±0.0404	0.7043±0.0972
BDPN [44]	8.9281±1.3466	7.1775±0.7231	0.9338±0.0329	0.7368±0.0869
DRPNN [31]	8.2857±1.1106	7.0249±0.8971	0.9369±0.0477	0.7500±0.0837
DiCNN [50]	7.9950±0.7276	7.1087±0.5381	0.9317±0.0465	0.6974±0.0891
FusionNet [51]	6.9218±1.1508	5.8424±0.6753	0.9363±0.0369	0.7472±0.0939
WINet [49]	5.2124±0.4128	4.2705±0.3069	0.9374±0.0311	0.7768±0.0885
SFINet [43]	5.4717±0.4181	4.5220±0.3691	0.9355±0.0306	0.7271±0.0919
PYDDN [13]	5.9936±0.4299	4.8959±0.2389	0.9323±0.0277	0.7476±0.1017
BIM [38]	5.2253±0.3598	4.2390±0.1307	0.9402±0.0202	0.7685±0.0937
FIAN(ours)	5.1602±1.1128	4.0046±0.9861	0.9455±0.0185	0.7858±0.1023
Ideal value	0	0	1	1

Average quantitative with the related standard deviations (std) comparisons on 20 reduced-resolution WorldView-2 examples, with the best performance highlighted in red.

**Fig 7:**
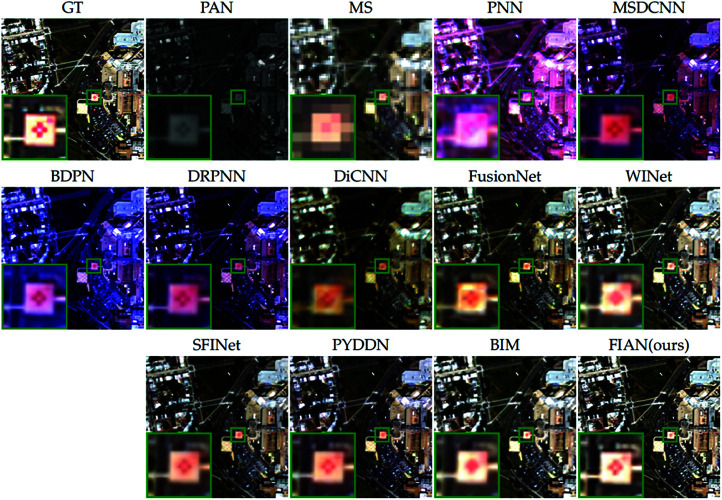
Visual comparisons of reduced-resolution WV2 data. The visual comparisons of fusion results from various methods applied to reduced-resolution data from the WV2 satellite. Reprinted from https://github.com/liangjiandeng/PanCollection under a CC BY license, with permission from Liangjian Deng, original copyright 2025.

Fig 7 provides a visual comparison of the pansharpening results from all compared methods to further illustrate the performance differences. We selected a representative area of the image (marked with a green box), enlarged it, and positioned it in the lower left corner of the original image for detailed observation. Close examination of the enlarged area reveals that the fusion results produced by our model closely resemble the ground truth image, exhibiting excellent performance in texture, edges, and color reproduction.

Secondly, we assess the pansharpening performance of various methods on actual data through full-resolution evaluation. Table 4 displays the objective assessment metric results for all compared methods and our proposed model across 20 full-resolution WV2 test samples. The results in the table demonstrate that our model achieves the best performance across all metrics, highlighting the effectiveness and superiority of our approach.

**Table 4:** Quantitative metrics of full-resolution WV2 data.

Method	QNR	$D_{\lambda }^{\left(K\right)}$	$D_{S}$
PNN [26]	0.7965±0.1270	0.1617±0.1072	0.0491±0.0351
MSDCNN [39]	0.6971±0.1062	0.2151±0.0690	0.1165±0.0580
BDPN [44]	0.6710±0.1270	0.2480±0.0835	0.1156±0.0706
DRPNN [31]	0.6923±0.1411	0.2638±0.1254	0.0943±0.0616
DiCNN [50]	0.6935±0.1108	0.2641±0.1002	0.0580±0.0323
FusionNet [51]	0.7960±0.1462	0.1571±0.1216	0.0603±0.0387
WINet [49]	0.8954±0.0614	0.0312±0.0208	0.0467±0.0436
SFINet [43]	0.8802±0.0956	0.0464±0.0310	0.0532±0.0493
PYDDN [13]	0.8872±0.0719	0.0425±0.0285	0.0451±0.0477
BIM [38]	0.8992±0.0731	0.0347±0.0398	0.0488±0.0389
FIAN(ours)	0.9045±0.0732	0.0304±0.0209	0.0438±0.0334
Ideal value	1	0	0

Average quantitative metrics across 20 full-resolution samples from the WorldView-2 dataset are presented, with the best performance highlighted in red.

**Fig 8:**
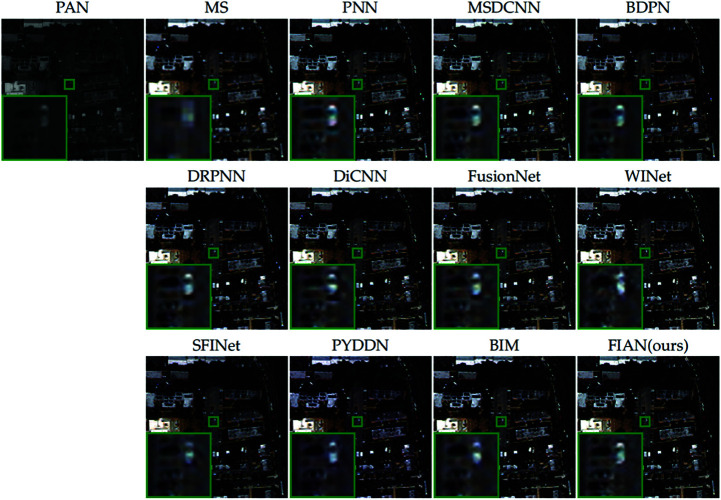
Visual comparisons of full-resolution WV2 data. The visual comparisons of fusion results from various methods applied to full-resolution data from the WV2 satellite. Reprinted from https://github.com/liangjiandeng/PanCollection under a CC BY license, with permission from Liangjian Deng, original copyright 2025.

Fig 8 provides a visual comparison of the pansharpening results from all methods evaluated, further illustrating performance differences. We selected a representative area of the image (marked with a green box), enlarged it, and placed it in the lower left corner of the original image for detailed observation. Upon close examination of the enlarged area, it is evident that the fusion results produced by our model excel in texture, edge definition, and color reproduction.

### Performance comparison with QB data

In this section, we conduct a comprehensive evaluation and comparison of the proposed method and benchmark methods on the QB dataset, considering both reduced-resolution and full-resolution aspects.

First, we assess the differences between the predicted pansharpening images and the ground truth (GT) images through reduced-resolution evaluation. Table 5 presents the objective assessment metric results for all compared methods and our proposed model across 20 WV2 reduce-resolution test samples. The table demonstrates that our model achieves the best performance across all metrics, showcasing the effectiveness and superiority of our approach. Compared to benchmark pansharpening methods, our model better preserves the spectral information of multispectral images while effectively enhancing spatial resolution, resulting in high-quality image fusion.

**Table 5:** Quantitative metrics of reduced-resolution QB data.

Method	SAM	ERGAS	SCC	Q2n
PNN [26]	10.8669±1.8058	9.4115±0.8517	0.8672±0.0241	0.7490±0.0339
MSDCNN [39]	7.8328±0.9019	8.3913±0.6621	0.8896±0.0183	0.7375±0.0207
BDPN [44]	8.5345±1.3053	8.5078±0.6433	0.8870±0.0202	0.7766±0.0219
DRPNN [31]	8.6753±1.0241	8.4124±0.5850	0.8971±0.0195	0.7861±0.0193
DiCNN [50]	11.1861±2.6916	10.6275±0.5739	0.8424±0.0242	0.7838±0.0316
FusionNet [51]	7.5344±1.1246	8.3390±0.7744	0.8884±0.0211	0.7707±0.0223
WINet [49]	4.8929±0.4929	3.9755±0.1544	0.9745±0.0093	0.8758±0.0072
SFINet [43]	5.0194±0.4918	4.0725±0.1353	0.9730±0.0099	0.8682±0.0077
PYDDN [13]	4.8812±0.4512	3.9362±0.1040	0.9747±0.0101	0.8735±0.0082
BIM [38]	5.0151±0.5203	4.0168±0.1214	0.9737±0.0096	0.8706±0.0082
FIAN(ours)	4.8586±0.4583	3.9267±0.1158	0.9749±0.0088	0.8788±0.0075
Ideal value	0	0	1	1

Average quantitative with the related standard deviations (std) comparisons on 20 reduced-resolution QuickBird examples, with the best performance highlighted in red.

**Fig 9:**
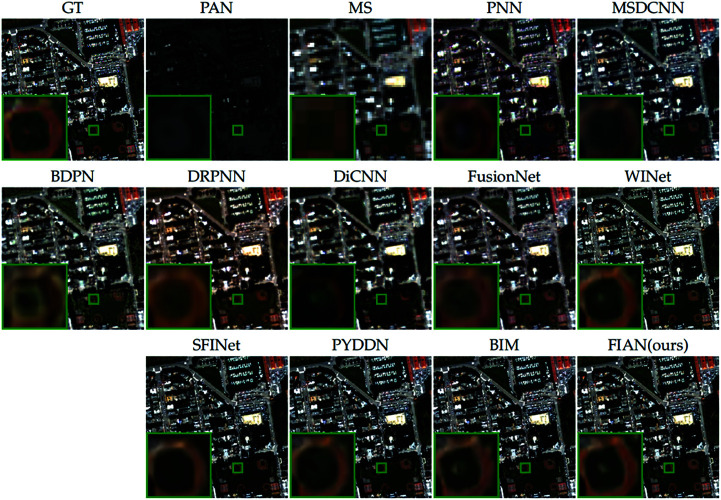
Visual comparisons of reduced-resolution QB data. The visual comparisons of fusion results from various methods applied to reduced-resolution data from the QB satellite. Reprinted from https://github.com/liangjiandeng/PanCollection under a CC BY license, with permission from Liangjian Deng, original copyright 2025.

Fig 9 provides a visual comparison of the pansharpening results from all compared methods to further illustrate the performance differences. We selected a representative area of the image (marked with a green box), enlarged it, and positioned it in the lower left corner of the original image for detailed observation. Close examination of the enlarged area reveals that the fusion results produced by our model closely resemble the ground truth image, exhibiting excellent performance in texture, edges, and color reproduction.

Secondly, we assess the pansharpening performance of various methods on actual data through full-resolution evaluation. Table 6 displays the objective assessment metric results for all compared methods and our proposed model across 20 full-resolution QB test samples. The results in the table demonstrate that our model achieves the best performance across all metrics, highlighting the effectiveness and superiority of our approach.

**Table 6:** Quantitative metrics of full-resolution QB data.

Method	QNR	$D_{\lambda }^{\left(K\right)}$	$D_{S}$
PNN [26]	0.8169±0.0660	0.1538±0.0586	0.0354±0.0220
MSDCNN [39]	0.8431±0.0493	0.1091±0.0514	0.0546±0.0117
BDPN [44]	0.7794±0.0489	0.1030±0.0374	0.1405±0.0270
DRPNN [31]	0.7883±0.0408	0.1126±0.0376	0.1093±0.0247
DiCNN [50]	0.8360±0.0549	0.1264±0.0532	0.0601±0.0241
FusionNet [51]	0.8590±0.0334	0.0792±0.0264	0.0690±0.0186
WINet [49]	0.9273±0.0240	0.0368±0.0160	0.0405±0.0140
SFINet [43]	0.9202±0.0236	0.0364±0.0110	0.0422±0.0153
PYDDN [13]	0.9200±0.0249	0.0436±0.0113	0.0429±0.0194
BIM [38]	0.9297±0.0188	0.0338±0.0130	0.0368±0.0116
FIAN(ours)	0.9337±0.0155	0.0313±0.0115	0.0299±0.0117
Ideal value	1	0	0

Average quantitative metrics across 20 full-resolution samples from the QuickBird dataset are presented, with the best performance highlighted in red.

**Fig 10:**
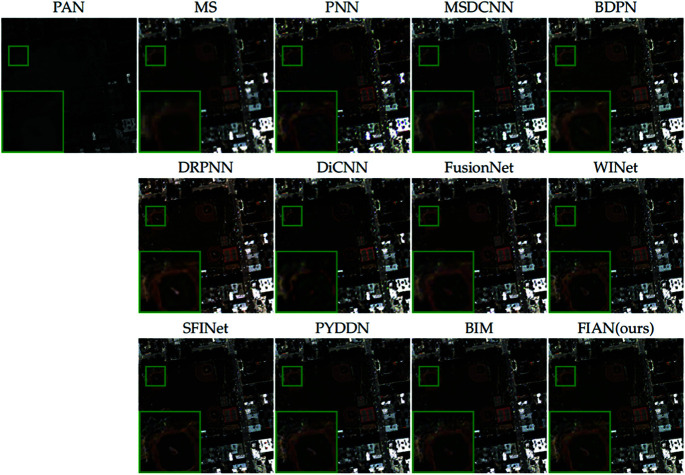
Visual comparisons of full-resolution QuickBird data. The visual comparisons of fusion results from various methods applied to full-resolution data from the QB satellite. Reprinted from https://github.com/liangjiandeng/PanCollection under a CC BY license, with permission from Liangjian Deng, original copyright 2025.

Fig 10 provides a visual comparison of the pansharpening results from all methods evaluated, further illustrating performance differences. We selected a representative area of the image (marked with a green box), enlarged it, and placed it in the lower left corner of the original image for detailed observation. Upon close examination of the enlarged area, it is evident that the fusion results produced by our model excel in texture, edge definition, and color reproduction.

### Ablation study

To further substantiate the efficacy of FIAN, we performed ablation experiments. This subsection delves into the importance of the frequency feature selection strategy, the multi-frequency information fusion module, and the frequency information-adaptive filter module.

Table 7 presents seven ablation experimental comparison strategies for the aforementioned modules. First, an ablation experiment was conducted on the frequency feature selection strategy. In FIAN, the last five network layers were selected as input for the frequency information-adaptive filter module by matching the gradient range of the ground truth (GT) with that of the nine-layer CNN network features. The ablation of the frequency feature selection strategy involved testing the impact of the first five and the last five CNN network layers on pansharpening performance, presented in Table 8 as (*I*), (*II*), (*III*), and (*IV*), with (*IV*) representing our proposed FIAN method.

**Table 7:** The ablation protocols for the suggested FIAN approach, in which the frequency information-adaptive filter module is denoted by FIAFM and the multi-frequency information fusion module is symbolized by MFIFM.

Config	The First five layers	The Last Five Layers	FIAFM	MFIFM
(*I*)	×	×	✓	✓
(*II*)	✓	✓	✓	✓
(*III*)	✓	×	✓	✓
(*IV*)	×	✓	✓	✓
(*V*)	×	✓	×	✓
(*VI*)	×	✓	✓	×
(*VII*)	×	✓	×	×

The ablation protocols for the suggested FIAN approach, in which the frequency information-adaptive filter module is denoted by FIAFM and the multi-frequency information fusion module is symbolized by MFIFM.

**Table 8:** Quantitative metrics of ablation methods on reduced-resolution WV3 data.

Method	SAM	ERGAS	SCC	Q2n
(*I*)	3.6418±0.3155	2.5963±0.3849	0.9741±0.0245	0.7953±0.0231
(*II*)	3.1991±0.3401	2.3480±0.3509	0.9729±0.0144	0.8193±0.0110
(*III*)	3.8411±0.3299	2.7415±0.4461	0.9678±0.0301	0.7553±0.0370
(*IV*)	3.1281±0.3099	2.2179±0.1884	0.9798±0.0082	0.8572±0.0097
(V)	3.1921±0.3700	2.3956±0.3828	0.9781±0.0117	0.8226±0.0138
(*VI*)	3.3385±0.4117	2.4983±0.4589	0.9778±0.0254	0.8193±0.0201
(*VII*)	3.6418±0.3155	2.5963±0.3849	0.9741±0.0345	0.7953±0.0331
Ideal value	0	0	1	1

Average quantitative metrics across 20 reduce-resolution samples from the WorldView-3 dataset are presented, with the best performance highlighted in red.

Following this, ablation experiments were conducted on the frequency information-adaptive filter module (FIAFM) and the multi-frequency information fusion module (MFIFM), precisely (*V*), (*VI*), and (*VII*). Notably, (*VII*) employs the same strategy as (*I*).

We conducted a series of ablation experiments on reduced-resolution data from the WorldView-3 satellite, with the objective metrics presented in Table 8. By comparing (*I*), (*II*), and (*III*) with (*IV*), we validated the effectiveness of the proposed frequency feature selection strategy; notably, (*IV*) achieved the best results across all metrics. The comparison between (*V*), (*VI*), and (*VII*) with (*IV*) further confirmed the efficacy of the frequency information-adaptive filter module (FIAFM) and the multi-frequency information fusion module (MFIFM).

**Fig 11:**
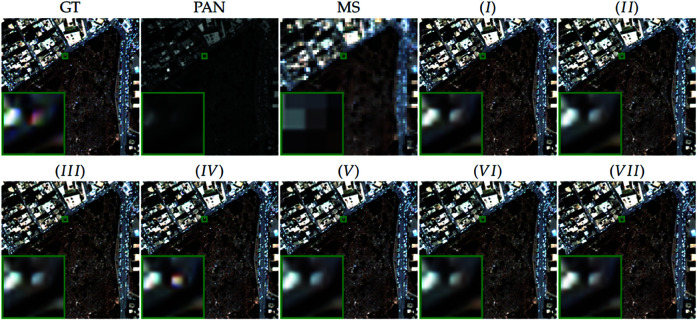
Visual comparisons of ablation methods on reduced-resolution WV3 data. Visual comparisons of the effects from all ablation methods applied to reduced-resolution data from the WorldView-3 satellite. Reprinted from https://github.com/liangjiandeng/PanCollection under a CC BY license, with permission from Liangjian Deng, original copyright 2025.

Fig 11 provides a visual comparison of the pansharpening results from all methods. Our proposed FIAN method (*IV*) most closely resembles the ground truth (GT) images, displaying superior spatial texture, edge clarity, and color reproduction compared to other scenarios.

## Conclusions

This study introduces the frequency information-adaptive network (FIAN), a groundbreaking pansharpening technique that seamlessly combines information from both the spatial and frequency domains. FIAN incorporates a frequency information-adaptive filter module that adaptively creates filters with varied frequency attributes by leveraging the gradients of distinct CNN features. FIAN also incorporates a frequency feature selection approach that aims to optimally complement and enhance high-frequency details while retaining low-frequency information. This targeted strategy ultimately elevates the overall performance of dual-domain information fusion by striking a balance between preserving crucial low-frequency components and enriching high-frequency nuances. Moreover, the multi-frequency information fusion module amalgamates data from diverse frequency ranges by executing a weighted combination of the amplitude and phase spectra, in conjunction with the spectral information derived from the LRMS image. This integrated approach ensures a comprehensive assimilation of information spanning the entire frequency spectrum, thereby enhancing the richness and fidelity of the fused output. Comprehensive evaluations performed on both downscaled and original resolution datasets showcase FIAN’s superior performance compared to cutting-edge pansharpening techniques. The proposed method shows better performance on quantitative metrics and produces visually appealing pansharpening images that preserve spatial details and spectral fidelity. While the proposed FIAN method demonstrates significant advantages in remote sensing pansharpening, the underlying adaptive frequency selection strategy can also be extended to other imaging domains, such as digital photography and image super-resolution.

Despite the demonstrated effectiveness of our method, certain limitations remain. First, the proposed neural network architecture is relatively complex, leading to longer training times and higher computational demands. Second, while the adaptive band-pass filtering strategy effectively suppresses noise in general, it currently employs a relatively simple model for frequency band selection. Future research could include more targeted modeling of noise characteristics to further enhance robustness. These limitations represent important directions for future improvements.
